# Indications for bi-cruciate retaining total knee replacement: An international survey of 346 knee surgeons

**DOI:** 10.1371/journal.pone.0234616

**Published:** 2020-06-15

**Authors:** Diarmuid De Faoite, Christian Ries, Michelle Foster, Christoph Kolja Boese

**Affiliations:** 1 Smith + Nephew, Baar, Switzerland; 2 Department of Orthopedics, University Medical Center Hamburg-Eppendorf, Hamburg, Germany; 3 Smith + Nephew, Hull, United Kingdom; 4 Department of Orthopaedic and Trauma Surgery, University Hospital of Cologne, Cologne, Germany; 5 Smith + Nephew GmbH, Hamburg, Germany; MD Anderson Cancer Center, UNITED STATES

## Abstract

There is limited evidence on total knee arthroplasty (TKA) that retains the anterior and the posterior cruciate ligaments. Bi-cruciate retaining (BCR) TKA is considered to show improved clinical function and kinematics. This survey aimed to (1) identify interest in and acceptance of BCR TKA surgery and (2) to capture the range of indications for BCR in the opinion of practicing knee surgeons. 346 surgeons with experience in TKA surgery completed a bi-lingual online survey. Demographics, arthroplasty experience as well as acceptance of and indications for BCR TKA were collected. 53 surgeons were experienced in BCR TKA and 225 would consider implanting it. A mean of 19.5% of TKA patients were considered eligible for BCR TKA. 56.3% were thought to have intact ACL at the time of TKA surgery. Surgeons were not likely to perform BCR TKA in patients with inflammatory arthritis, aged over 80, BMI above 34.9 kg/m^2^, a varus or valgus deformity of more than 10° and flexion contractures of more than 10°. There is strong interest among orthopedic surgeons to perform BCR TKA and the percentage of potentially eligible patients is high. Significant joint deformity is a limitation of BCR TKA, while age and high BMI are less relevant. BCR TKA experienced surgeons are less restrictive.

## Introduction

Most total knee arthroplasty (TKA) techniques involve sacrificing the anterior cruciate ligament or both cruciate ligaments.[1, 2] Few TKA systems allow for the preservation of both cruciate ligaments.[2, 3] TKA that retains the posterior cruciate ligament (PCL) as well as the anterior cruciate ligament (ACL) is referred to as bi-cruciate retaining (BCR) TKA.[3–5]

Functionally intact cruciate ligaments are required for BCR TKA. Studies show that up to 78% of ACLs are intact at the time of the TKA.[6, 7] However, BCR TKAs are seldom implanted and few implant systems are available.[3] While it is assumed that retaining the ACL may result in more natural knee kinematics contributing to higher patient satisfaction,[2, 3, 8] there are concerns about technical difficulties, complication risks, long term survival and functional outcomes.[3, 9, 10] A systematic literature review of the available BCR TKA clinical evidence revealed outcomes comparable to other TKA designs.[3] However, there is a lack of knowledge on the relevant indications for BCR TKA, and most importantly, the limitations to indications.

This survey aimed to (1) identify interest in and acceptance of BCR surgery and (2) to capture the range of indications for BCR in the opinion of practicing knee surgeons.

## Materials and methods

A bi-lingual (German and English) questionnaire was developed. The questionnaire was published online through SurveyMonkey™ (San Mateo, CA, US). Questions posed included information about demographics, arthroplasty experience as well as acceptance of and indications for BCR TKA.

From October 2018 to March 2019, surgeons were contacted personally by the authors or by e-mail using the internal GDPR-compliant distribution list of healthcare professionals. All communication was aligned with global and local laws, as well as privacy and data protection regulations. The ethics commission (IRB) granted a waiver as long as no personal identifiers were collected as part of the study (Ethikkommission der Ärztekammer, Hamburg, Germany). No personal identifiers were collected. All participants electronically agreed to the terms and conditions.

### Survey design

The survey design was informed by unstructured interviews with senior knee surgeons (n = 11) and a systematic literature review on BCR TKA. The draft survey was circulated to a team of three in-house orthopedic surgeons and the clinical, scientific and medical affairs team for content development. Repeated circulation of modified survey versions was performed until consensus was found. Due to the use of skip logic in the questionnaire, not all questions were answered by each respondent.

The survey was available in two languages (English, German). Native speakers of both languages performed forward and backward translations until consensus was found. The survey consisted of 39 questions, the final being an open ended comment field. The complete questionnaires in both languages are available as supplemental material (S1 File).

Inclusion criteria required respondents to be a) a medical doctor (MD) with experience in orthopedic surgery and b) with experience with TKA. Inexperience with TKA or any non-doctors were therefore excluded from the survey.

Incomplete surveys were eliminated from the analysis per protocol. Contradictory answers to specific questions of completed surveys were ignored. Free text comments were stratified into specific answer categories for analysis where appropriate.

### Stratification

#### Answers given as percentages

Stratification into groups of 20% increments was performed (e.g. 1–20%, 21–40%, etc.).

#### Age

The upper age limitations considered appropriate for BCR TKA indication were stratified into groups of <45 years (young), 45–64 years (middle-aged), 65–79 years (aged) and 80 years or older using the Medical Subject Headings (MeSH) classification.[11]

#### BMI

The upper body mass index (BMI) limitations considered appropriate for BCR TKA indication were stratified into groups using the World Health Organization (WHO) classification.[12] The categories used were underweight (<18.5 kg/m^2^), normal weight (18.5–24.9 kg/m^2^), pre-obesity (25–29.9 kg/m^2^), obesity class I (30–34.9 kg/m^2^), obesity class II (35–39.9 kg/m^2^) and obesity class III (40+ kg/m^2^).

#### Statistical analysis

The results from the German and English language surveys were pooled into one dataset. A senior biostatistician (MF) conducted the statistical analysis. Data output from SurveyMonkey was exported as a Microsoft Excel file (Microsoft Office Professional Plus 2016, Microsoft Corp., Redmond, WA, USA). The data analysis for this paper was generated using SAS software, (Version 9.4., SAS Institute Inc., Cary, NS, USA).

Continuous variables were summarized with the following summary statistics: number of observations, mean, median, standard deviation, minimum and maximum values. Categorical variables were summarized as frequencies and percentages. Histograms and box-plots were used to visualize data. Unless otherwise stated, all significance tests and hypothesis testing were two-sided, performed at the 5% significance level. All variables were tested for normality using a Shapiro-Wilks normality test and non-parametric tests were applied where appropriate. Where statistical significance was achieved, post-hoc tests were used and Bonferroni corrections were applied to adjust for the number of all possible pairs. Spearman’s Rank correlation coefficients were calculated for correlation analysis.

## Results

### Baseline characteristics of respondents

346 completed surveys were collected and analyzed. 82 (23.7%) were from the German language survey and 264 (76.3%) from the English language survey. Baseline demographic characteristics of the respondents are displayed in **Table 1**. Information on arthroplasty experience is presented in **S1 File.**

**Table 1:** Baseline characteristics.

	Answer	N (%)
**Sex**	Male	332 (96.0%)
	Female	12 (3.4%)
	Prefer not to say	2 (0.6%)
**Position**	Attending Physician	279 (80.7%)
	Fellow	26 (7.6%)
	Resident	21 (6.0%)
	Other	20 (5.8%)

German job titles have been amalgamated. Attending physician includes *Chefarzt* and *Oberarzt*. *Facharzt* is Fellow. *Weiterbildungsassistent* is resident.

### Key outcomes

53 surgeons (15.3%) had operative experience with BCR implants. The most commonly used BCR implants were Journey^TM^ II XR (Smith + Nephew plc., Watford, UK) (n = 38; 71.7%), Vanguard XP^TM^ (Zimmer Biomet, Warsaw, Indiana, USA) (n = 15; 28.3%) and others (n = 19; 35.8%).

225 (65.0%) of surgeons would consider implanting a BCR total knee prosthesis; 65 (18.8%) were not sure; and 56 (16.2%) would not.

135 surgeons (39.0%) always sacrifice the PCL during TKA surgery, 140 surgeons (40.5%) sometimes sacrifice the PCL and 34 surgeons (9.8%) never sacrifice the PCL. 37 respondents (10.7%) did not record a response to this question.

A mean of 19.5% (median 13, SD 19.99; range 0–80) of patients indicated for a knee replacement were deemed suitable for BCR (**Table 2**).

**Table 2:** Percentage of suitability for BCR.

Percentage of patients suitable for BCR implant		Do you sacrifice the PCL during TKA procedures?^1^				Would you consider implanting a BCR total knee prosthesis?			
	N (%)	Always	Some-times	Never	Total	Yes	No	I do not know	Total
None	61 (17.6%)	32	18	10	**60**	17	28	16	**61**
1–20%	173 (50.0%)	65	73	16	**154**	115	24	34	**173**
21–40%	58 (16.8%)	23	25	3	**51**	45	2	11	**58**
41–60%	37 (10.7%)	9	18	3	**30**	34	0	3	**37**
61–80%	17 (4.9%)	6	6	2	**14**	14	2	1	**17**
81–100%	0 (0%)	0	0	0	**0**	0	0	0	**0**
**Total**	**346**	**135**	**140**	**34**	**309**	**225**	**56**	**65**	**346**

^1^ 37 missing responses.

There was a statistically significant difference in the percentage of patients suitable for BCR TKA implantation between surgeons’ preference of sacrificing the PCL (i.e. always, sometimes, never) during TKA procedures (p = 0.0095). Surgeons sometimes sacrificing the PCL were most likely to indicate BCR TKA (“sometimes” vs. “always”: p = 0.0327; “sometimes” vs. “never”: p = 0.0516).

Surgeons with experience with partial knee arthroplasty (PKA) were more likely to indicate BCR TKA compared to those without PKA experience (p = 0.0041). The willingness to indicate BCR TKA showed significant differences between surgeons’ experiences with different types of PKA (**Table 3**).

**Table 3:** Experience in partial knee arthroplasty.

	Percentage of patients indicated for a knee replacement suitable for BCR											
	Experience with PKA					No experience with PKA						
	N	Mean	Median	SD	Range	N	Mean	Median	SD	Range	Z score	p-value^1^
All Partial Knees	274	20.5	15	19.87	0–80	72	15.5	6	20.07	0–80	-2.871	0.0041
UKA	240	20.1	15	19.48	0–80	106	18.0	10	21.13	0–80	-1.781	0.2250^2^
PJF	114	20.2	15	19.54	0–80	232	19.1	10	20.24	0–80	1.007	0.9417^2^
Bicompartmental PKA	83	25.5	20	20.65	0–80	263	17.6	10	19.43	0–80	3.919	0.0003^2^

^1^ Wilcoxon Signed Rank test

^2^ Bonferroni correction applied

Surgeons reported the percentage of patients presenting with a functionally intact ACL at the time of TKA surgery to be 56.3% (median 60, SD 23.01; range 0–100) (**S2 File and S3 File**). Participants would apply MRI (67%, n = 232) or a pre-operative clinical function test (90%, n = 311) of the ACL to determine the ACL status (**S4 File**). 136 participants (39%) would indicate BCR TKA in ACL with signs of degeneration (**S4 File)**.

**Fig 1:**
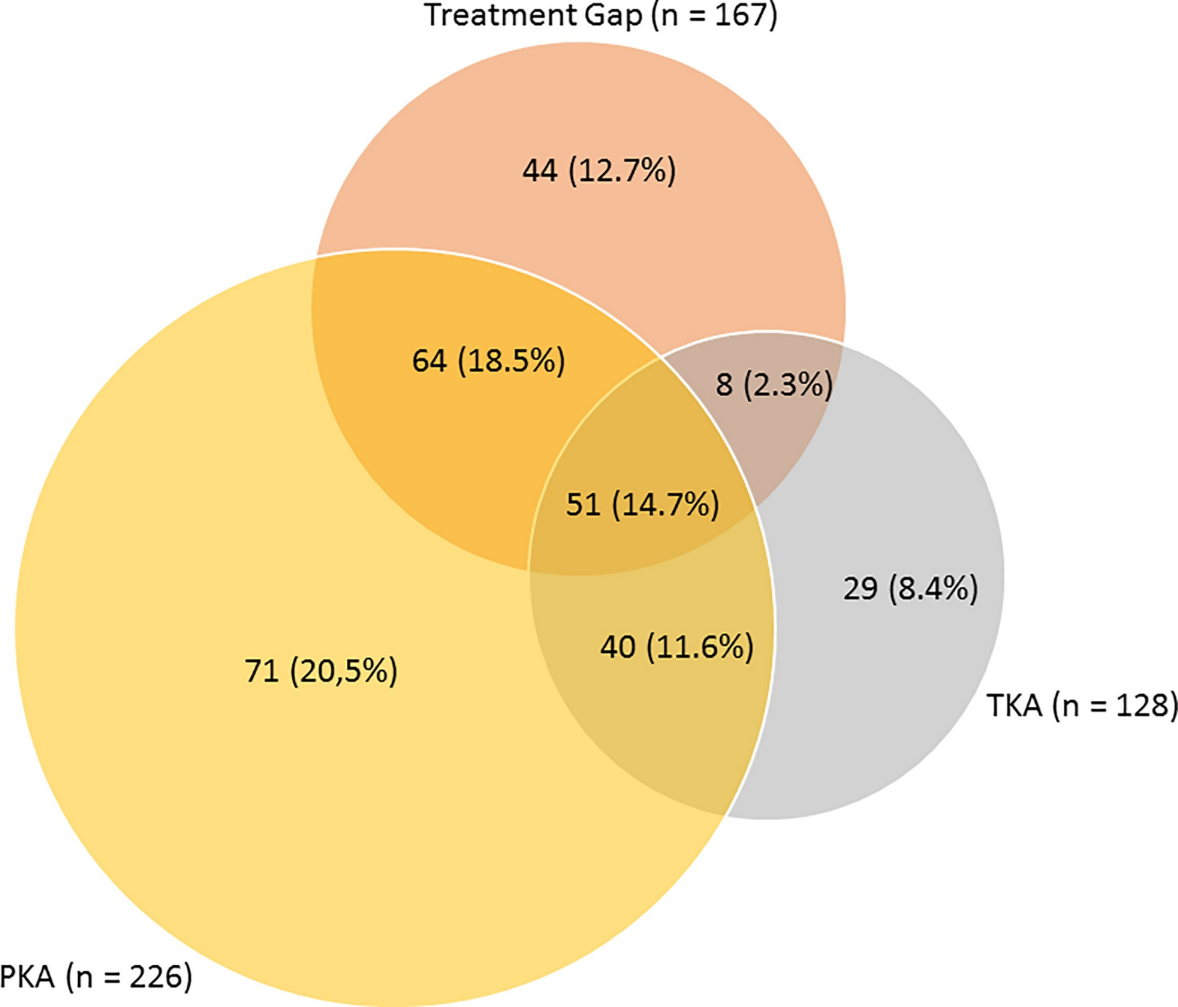
Multiple choice selection of indications for BCR TKA. Options were same indication as (1) partial knee arthroplasty (PKA), (2) total knee arthroplasty (TKA) or a treatment gap between the two.

The BCR indication spectrum overlapped with indication for PKA, TKA or a treatment gap between PKA and TKA (**Fig 1**).

### Indications

#### Age

167 (48.3%) surgeons considered age a relevant indication factor. 153 (91.6%) defined an upper age limit. The mean limit was 68.0 years (n = 153; median 70; SD 9.82; range 36–99) (**Table 4**).

**Table 4:** Indication age. Stratified by MeSH groups.

	Upper limit	
Age group	N	%
< 45	2	1.3%
Middle aged (45–64)	49	32.0%
Aged (65–79)	82	53.6%
80 or more	20	13.0%
Total	153	100%

179 surgeons did not answer this question.

#### BMI

179 (51.7%) surgeons found BMI to be a relevant factor for BCR TKA indication. Of these, 177 (98.9%) defined an upper limit. The mean limit was 32.6 kg/m^2^ (n = 177; median 34; SD 4.26; range 25–40) (**Table 5**).

**Table 5:** Indication BMI. Stratification by WHO categories.

	Upper limit	
BMI (kg/m^2^)	N	%
Less than 18.5	0	0%
18.5–24.9	0	0%
25–29.9	33	18.7%
30–34.9	62	35.0%
35–39.9	63	35.6%
40 +	19	10.8%
Total	177	100%

167 surgeons did not answer this question.

#### Contracture

**Fig 2:**
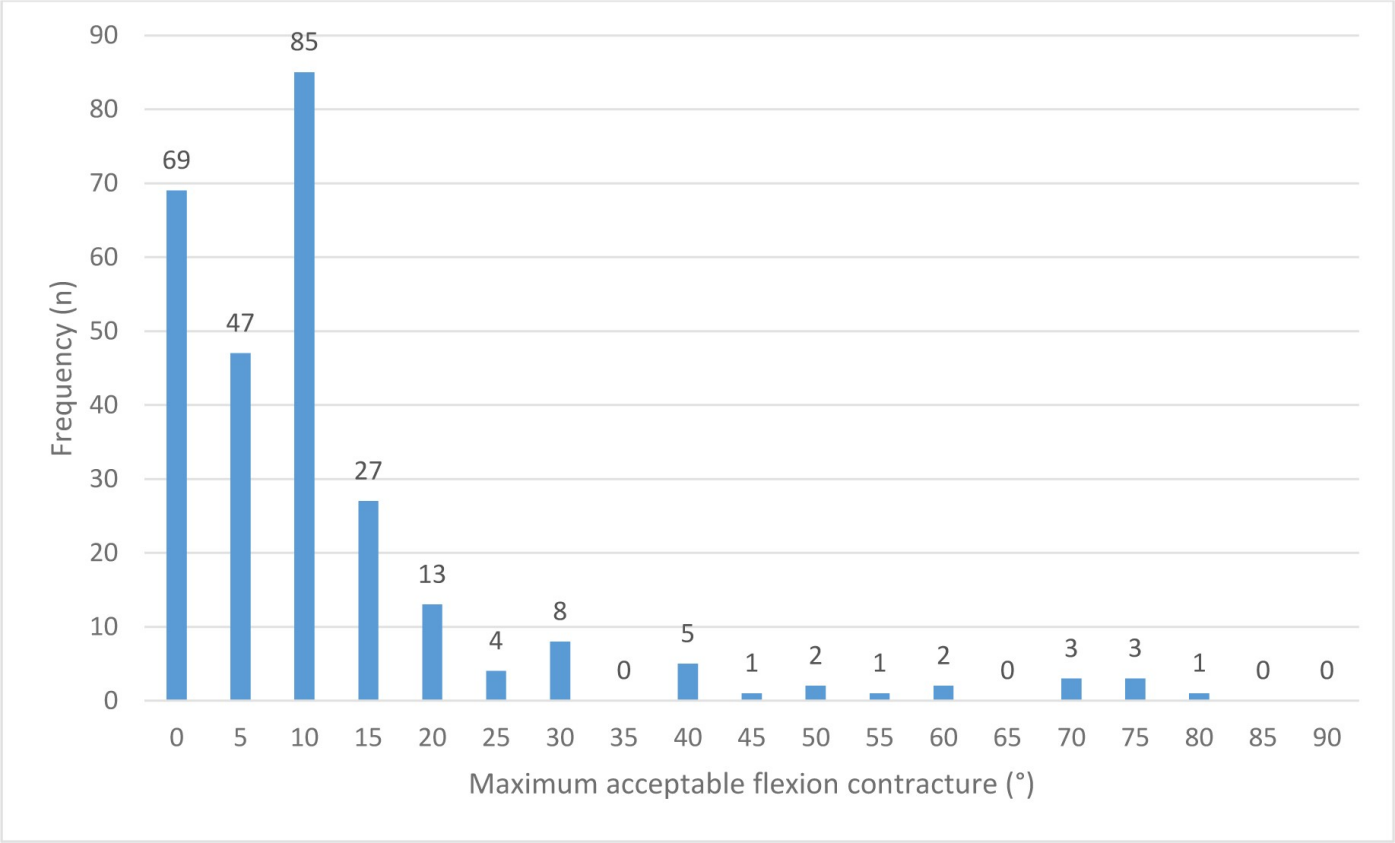
Diagram of acceptable maximum contractures.

278 (80.3%) surgeons considered flexion contracture to be a relevant factor for BCR TKA indication. Contracture was reported as maximum acceptable degrees. 7 (2.5%) respondents selected a maximum of 90° defined as “no limitation”. Excluding answers without limits, the mean limit was 11.5° (n = 271; median 10; SD 14.64; range 0–80) (**Fig 2**).

#### Deformity

268 (77.5%) surgeons deemed deformities a relevant factor for BCR TKA indication. Of these, 251 (93.7%) would accept varus deformities with a mean of 11.9° (n = 251; median 10; SD 5.91; range 5–35). 236 (88.0%) respondents would accept valgus deformities with a mean of 10.6° (n = 236; median 10; SD 5.16; range 5–35). 254 (94.8%) surgeons answered the acceptable posterior slope was limited to a mean of 9.4° (n = 254; median 10; SD 4.44; range 5–40) (**Table 6**).

**Table 6:** Indication deformity.

Degree	Varus		Valgus		Slope	
°	N	%	N	%	N	%
0	17	6.3	32	11.9	13	4.9
5	51	19.0	63	23.5	84	31.3
10	115	42.9	113	42.2	132	49.3
15	46	17.2	43	16.0	29	10.8
20	26	9.7	10	3.7	5	1.9
25	6	2.2	2	0.8	2	0.8
30	5	1.9	4	1.5	1	0.4
35	2	0.8	1	0.4	0	0
40	0	0	0	0	1	0.4
45	0	0	0	0	1	0.4
Total	268	100%	268	100%	268	100%
Mean	11.1°		9.3°		9.1°	

78 surgeons did not answer this question.

Willingness or unwillingness to indicate BCR TKA did not influence the distribution of indication ranges. Surgeons’ preference for PCL sacrifice only affected upper BMI as limitations to indications (p = 0.0274). P-values are provided for every indication category in **Tables 7 and 8.**

**Table 7:** Indication summary vs. BCR indication and PCL sacrifice.

	Do you sacrifice the PCL during TKA procedures?															
	Always					Sometimes					Never					
	N	Mean	Median	SD	Range	N	Mean	Median	SD	Range	N	Mean	Median	SD	Range	p-value^1^
Age	70	66.7	65	10.50	36–99	62	69.0	70	9.66	47–90	14	68.3	70	8.14	55–82	0.3198
BMI	70	32.1	32	4.34	25–40	77	32.4	33	4.18	25–40	15	35.5	35	4.03	28–40	0.0274
Contracture	101	11.8	10	14.79	0–80	112	12.6	10	16.18	0–75	24	9.4	5	15.42	0–75	0.4693
Varus	88	12.4	10	6.21	5–30	111	11.7	10	5.37	5–30	17	12.6	10	7.93	5–35	0.8079
Valgus	84	11.1	10	5.17	5–30	106	10.6	10	5.29	5–35	15	12.3	10	6.23	5–30	0.4313
Slope	89	9.5	10	4.33	5–30	113	9.7	10	5.01	5–40	19	8.4	10	2.39	5–10	0.7226

^1^ Kruskal Wallis test.

37 missing responses.

**Table 8:** Indication summary vs. BCR indication and general consideration of BCR TKA.

	Would you consider implanting a BCR total knee prosthesis?															
	Yes					No					Don’t know					
	N	Mean	Median	SD	Range	N	Mean	Median	SD	Range	N	Mean	Median	SD	Range	p-value^1^
Age	106	68.4	70	9.10	40–99	19	66.5	65	13.18	50–90	28	67.1	65.5	10.06	36–85	0.4202
BMI	127	32.9	34	4.13	25–40	21	31.7	30	4.70	25–40	29	31.6	31	4.39	25–40	0.2374
Contracture	189	13.2	10	15.89	0–80	36	4.6	0	5.90	0–20	46	10.1	10	12.27	0–75	0.0002
Varus	179	12.3	10	5.91	5–35	29	10.2	10	5.74	5–30	43	11.3	10	5.89	5–35	0.0813
Valgus	173	10.9	10	5.25	5–35	23	9.6	10	6.20	5–30	40	9.8	10	3.91	5–20	0.1535
Slope	181	9.4	10	3.86	5–25	31	9.7	10	5.31	5–30	42	9.4	10	5.97	5–40	0.7008

1: Kruskal Wallis test

## Discussion

This survey collected the opinions of practicing knee surgeons on BCR TKA. The primary aim was characterization of interest in and acceptance of BCR surgery. Secondary goals were descriptions of perceived ranges and limitations of indications for BCR TKA. Overall, 346 surgeons with experience in knee arthroplasty completed the survey. The respondents covered a wide range of experience including high, medium and low volume knee arthroplasty. Only 3.4% were female and 88.3% were attending physicians or fellows with extensive experience. Notably, a relevant number of surgeons also had experience with PKA but only 15.3% had previously performed a BCR TKA.

There was high interest in performing BCR TKA. 65.0% of the participants would consider implanting a BCR TKA and another 18.8% were not sure. Only a few surgeons would not consider it. Additionally, 222 (64.2%) surgeons said there is a need for BCR TKA.

Despite this strong interest in BCR TKA, there were concerns regarding the technical difficulty of the procedure. While this is partially supported by existing literature,[13] there is no clear indication of more complications.[3] Although Christensen et al. found higher revision rates for a modern BCR TKA after 18 months follow up,[13] this finding could not be supported by Baumann et al. and Alnachoukati et al. for the same implant.[2, 9, 14] Notably, older implants show good long-term survival up to 24 years.[4, 5]

There is a significant lack of knowledge around patient segmentation for BCR TKA. Most publications on BCR TKA do not specify limitations to indications.[2, 4, 5, 9, 13, 15]

Interestingly, the answers received indicated that BCR TKA could be an alternative to PKA. Possible reasons might be the combination of expected advantages of both procedures: the more natural knee kinematics achieved with UKA or bicompartmental arthroplasty and long-term survival of TKA. This might also be a reason for the higher patient satisfaction expected after BCR TKA. Johnson recorded as few as 7% of TKA patients reporting that their knee feels normal after surgery[6] while Alnachoukati et al. report 91% of BCR patients saying their knee always or sometimes feels normal.[14] Survival analysis of old BCR TKA designs with follow-up of up to 24 years reached 89% (82–93%) in a study by Pritchett. For modern designs, the follow-up times were shorter and results were heterogeneous. While Alnachoukati et al. reported 1.4% at 12 months,[16] Baumann et al. found revision rates of up to 5.9% at 18 months and,[9] Pelt et al. 17% revisions for any reason at 36 months for the same implant type.[17] Baumann et al. directly compared UKA, BCR TKA and standard TKA. They found BCR TKA and UKA to have comparable joint awareness in terms of the forgotten joint score (BCR TKA 53.4 vs. UKA 53.6 points). Both were superior to the standard TKA group (38.9 points) yet no objective knee scores were reported.[2, 9]

There is consensus regarding the importance of the ACL for natural knee kinematics and proprioception. A functionally intact ACL is a prerequisite for implantation of BCR TKA. In our study, surgeons reported an average of over 55% of patients undergoing TKA to have an intact ACL. This is supported by Johnson et al. and a current systematic literature review on the subject.[6, 18] Notably, Cloutier et al. did not find ACL degeneration to influence survival and clinical outcomes of BCR TKA in the long term.[19] In this survey, the majority of surgeons (90%) considered pre-interventional clinical function tests and 67% MRI to be to be reasonable.

While BCR TKA is often said to be developed for young and active patients, the literature shows wide ranges of age indicated for BCR TKA. In this survey, less than 50% of respondents considered patient age to be relevant. The mean upper age limit was set at around 68 years. While few surgeons would indicate BCR TKA in patients above 79 years of age, only about 9% would limit the indication to patients younger than 60 years. Overall, there was no clear trend toward excluding older patients.

It has been reported that BMI may be linked to complications and survival of TKA implants.[20] However, Baumann did not find a BMI influence on patient-reported outcomes and survival. In this survey, 47.5% of the knee surgeons considered high BMI to be a relevant factor for indication. While 18.7% limited BMI to pre-obesity, most would accept Class I and II obesity. However, only 10.8% would implant BCR TKA in patients with Class III obesity and 53.7% would not indicate above Class I. This is in line with current guidelines on TKA indications.[21]

Christensen et al. and Pritchett et al. restricted BCR TKA indication to minimal or limited flexion contractures.[13, 22] The limited possibility to address reasons for contracture during BCR TKA surgery may be the rationale for this. The majority of survey participants (n = 268) considered contractures relevant contraindications. 25.5% would not accept any contracture; 17.3% no more than 5° and 31.4% would accept up to 10°. Only 15.9% accepted contractures of more than 15° (**Fig 2**).

Similar to contractures, the degree of deformity might limit the indication for BCR TKA. In most publications on BCR TKA, there were limitations to valgus deformity of maximum 10–30° and varus deformity of up to 10–30°.[2, 15, 22] Underlying reasons may have been the assumption of impaired ACL function in severe deformities as well as impaired collateral ligament impairment. In this survey, restrictions to varus and valgus deformity were quite similar. Slightly less valgus deformity was accepted. The majority of surgeons would not indicate BCR TKA in more than 10° of varus (68.2%) or valgus (77.6%) deformity. These limitations may reflect the lack of constraint in BCR TKA which reduce the options of soft tissue balancing.

Interestingly, except for acceptable contractures, there were no differences regarding limitations between surgeons willing to perform BCR TKA or not. Similarly, PCL sacrifice preferences did not influence indication ranges. Only the upper BMI limit showed differences between the groups. However, previous experience with BCR TKA influenced the limitations regarding upper BMI, upper age, contractures and varus deformity. In all cases, experienced surgeons were less restrictive.

Of note, the number of patients considered eligible for BCR TKA showed significant differences between surgeons’ PCL sacrifice preferences. Surgeons without strict PCL sacrifice regimes (“sometimes” vs. “always” or “never”) were more likely to perform BCR TKA. This may indicate the openness of such surgeons to perform patient-specific procedures.

### Limitations

Firstly, the survey was distributed by an electronic mailing list that included non-knee surgeons. Therefore, the response rate from eligible candidates could not be calculated. However, the pre-study aim of at least 100 completed surveys for analysis was overachieved.

Secondly, most surgeons had no prior experience with BCR TKA. Therefore, this study represents opinions and assumptions. Still, these subjective opinions contain valuable information and help to better understand the potential role of BCR TKA in the spectrum of knee replacements. Additionally, the training and individual background of surgeons may affect answers. Specific differences between sports and joint fellowship trained physicians were not possible in this study.

Thirdly, the survey was only available in English and German. The perception towards BCR TKA might vary between cultures and multi-language surveys might be able to detect such variances.

However, despite these limitations, this survey is the first large scale sampling of surgeons’ opinions of bi-cruciate retaining surgery.

## Conclusions

There is strong interest from orthopedic surgeons to perform BCR TKA. About 20% of their TKA patients may be eligible for BCR TKA. The survey showed the majority (75% or more) of surgeons would perform BCR TKA in patients up to an age of 80 years and with a deformity of up to 10° varus or valgus. Flexion contractures of up to 10° are acceptable to most of the survey respondents. BCR TKA for a patient with a BMI above 34.9 kg/m^2^ is supported by few surgeons. BCR TKA experienced surgeons are less restrictive.

## Supporting information

S1 File English and German survey. Complete questionnaire EXCEL file.
(XLSX)

S2 File Experience of participating surgeons. Supplemental table.
(DOCX)

S3 File ACL intactness and suitability for BCR implantation. Supplemental table.
(DOCX)

S4 File ACL status. (DOCX)

S5 File Individual answers. Excel file with complete dataset.
(XLSX)

## References

[1] Parcells BW, Tria AJ Jr. The Cruciate Ligaments in Total Knee Arthroplasty. American journal of orthopedics (Belle Mead, NJ). 2016;45(4):E153–60. 27327919

[2] Baumann F, Bahadin O, Krutsch W, Zellner J, Nerlich M, Angele P, et al Proprioception after bicruciate-retaining total knee arthroplasty is comparable to unicompartmental knee arthroplasty. Knee surgery, sports traumatology, arthroscopy: official journal of the ESSKA. 2017;25(6):1697–704. 10.1007/s00167-016-4121-2 27145774

[3] Cherian JJ, Kapadia BH, Banerjee S, Jauregui JJ, Harwin SF, Mont MA. Bicruciate-retaining total knee arthroplasty: a review. The journal of knee surgery. 2014;27(3):199–205. 10.1055/s-0034-1374812 24764232

[4] Sabouret P, Lavoie F, Cloutier JM. Total knee replacement with retention of both cruciate ligaments: a 22-year follow-up study. The bone & joint journal. 2013;95-b(7):917–22. 23814243 10.1302/0301-620X.95B7.30904

[5] Pritchett JW. Bicruciate-retaining Total Knee Replacement Provides Satisfactory Function and Implant Survivorship at 23 Years. Clinical orthopaedics and related research. 2015;473(7):2327–33. 10.1007/s11999-015-4219-8 25721577 PMC4457764

[6] Johnson AJ, Howell SM, Costa CR, Mont MA. The ACL in the arthritic knee: how often is it present and can preoperative tests predict its presence? Clinical orthopaedics and related research. 2013;471(1):181–8. 10.1007/s11999-012-2505-2 22864617 PMC3528932

[7] Abdelbadie A, Toreih AA, Radwan MA. ACL status in arthroplasty patients, why not to preserve? Sicot-j. 2018;4:1 10.1051/sicotj/2017042 29309029 PMC5757387

[8] Komistek RD, Allain J, Anderson DT, Dennis DA, Goutallier D. In vivo kinematics for subjects with and without an anterior cruciate ligament. Clinical orthopaedics and related research. 2002(404):315–25. 10.1097/00003086-200211000-00047 12439275

[9] Baumann F, Krutsch W, Worlicek M, Kerschbaum M, Zellner J, Schmitz P, et al Reduced joint-awareness in bicruciate-retaining total knee arthroplasty compared to cruciate-sacrificing total knee arthroplasty. Archives of orthopaedic and trauma surgery. 2018;138(2):273–9. 10.1007/s00402-017-2839-z 29124363

[10] Jenny JY, Jenny G. Preservation of anterior cruciate ligament in total knee arthroplasty. Archives of orthopaedic and trauma surgery. 1998;118(3):145–8. 10.1007/s004020050335 9932189

[11] Geifman N, Cohen R, Rubin E. Redefining meaningful age groups in the context of disease. Age (Dordr). 2013;35(6):2357–66. 10.1007/s11357-013-9510-6 23354682 PMC3825015

[12] WorldHealthOrganization. http://www.euro.who.int/en/health-topics/disease-prevention/nutrition/a-healthy-lifestyle/body-mass-index-bmi: WHO; [

[13] Christensen JC, Brothers J, Stoddard GJ, Anderson MB, Pelt CE, Gililland JM, et al Higher Frequency of Reoperation With a New Bicruciate-retaining Total Knee Arthroplasty. Clinical orthopaedics and related research. 2017;475(1):62–9. 10.1007/s11999-016-4812-5 27043727 PMC5174026

[14] Alnachoukati OK, Emerson RH, Diaz E, Ruchaud E, Ennin KA. Modern Day Bicruciate-Retaining Total Knee Arthroplasty: A Short-Term Review of 146 Knees. J Arthroplasty. 2018. 10.1016/j.arth.2018.03.026 29666029

[15] Cloutier JM. Long-term results after nonconstrained total knee arthroplasty. Clin Orthop Relat Res. 1991(273):63–5. 1959288

[16] Alnachoukati OK, Emerson RH, Diaz E, Ruchaud E, Ennin KA. Modern Day Bicruciate-Retaining Total Knee Arthroplasty: A Short-Term Review of 146 Knees. The Journal of arthroplasty. 2018;33(8):2485–90. 10.1016/j.arth.2018.03.026 29666029

[17] Pelt CE, Sandifer PA, Gililland JM, Anderson MB, Peters CL. Mean Three-Year Survivorship of a New Bicruciate-Retaining Total Knee Arthroplasty: Are Revisions Still Higher Than Expected? The Journal of arthroplasty. 2019;34(9):1957–62. 10.1016/j.arth.2019.04.030 31160154

[18] Roussi K, Saunders C, Boese CK, J. W, editors. Intactness of the Anterior Cruciate Ligament at Time of Total Knee Arthroplasty Surgery in Patients With Osteoarthritis: A Systematic Literature Review and Meta-Analysis. 32nd Annual Congress of the International Society for Technology in Arthroplasty; 2019; Toronto: International Society for Technology in Arthroplasty (ISTA)

[19] Cloutier JM, Sabouret P, Deghrar A. Total knee arthroplasty with retention of both cruciate ligaments. A nine to eleven-year follow-up study. The Journal of bone and joint surgery American volume. 1999;81(5):697–702. 10.2106/00004623-199905000-00011 10360698

[20] McElroy MJ, Pivec R, Issa K, Harwin SF, Mont MA. The effects of obesity and morbid obesity on outcomes in TKA. J Knee Surg. 2013;26(2):83–8. 10.1055/s-0033-1341407 23479424

[21] Lutzner J, Lange T, Schmitt J, Kopkow C, Aringer M, Dgrh, et al [The S2k guideline: Indications for knee endoprosthesis: Evidence and consent-based indications for total knee arthroplasty]. Orthopade. 2018;47(9):777–81. 10.1007/s00132-018-3612-x 30097685

[22] Pritchett JW. Patient preferences in knee prostheses. The Journal of bone and joint surgery British volume. 2004;86(7):979–82. 10.1302/0301-620x.86b7.14991 15446522

