# Supplementary material for: Indications for bi-cruciate retaining total knee replacement: An international survey of 346 knee surgeons
Source: PLoS One. 2020 Jun 15;15(6):e0234616. doi: 10.1371/journal.pone.0234616 (PMC7295230; doi:10.1371/journal.pone.0234616)
Supplement: S2 File — Supplemental table. (DOCX) [file pone.0234616.s002.docx]

**Digital Supplemental Content 2**

**Experience of participating surgeons.**

HCPs could select multiple answers for the partial knee categories.

|  | **Answers (n)** | **Mean** | **SD** | **Median** | **Min** | **Max** |
| --- | --- | --- | --- | --- | --- | --- |
| **Years of Experience TKA (total)** | 346 | 16.2 | 8.89 | 15 | 1 | 50 |
| **Procedures per year** |  |  |  |  |  |  |
| **Primary TKA** | 346 | 133.3 | 130.38 | 100 | 10 | 1000 |
| **Revision TKA** | 272 | 22.2 | 17.81 | 20 | 1 | 110 |
| **Partial knee** | 274 |  |  |  |  |  |
| - Unicompartmental knee arthroplasty | 229 | 24.7 | 28.52 | 15 | 2 | 200 |
| - Bicompartmental knee arthroplasty | 69 | 48.0 | 48.27 | 30 | 5 | 200 |
| - Patellofemoral joint replacement | 93 | 9.1 | 13.82 | 5 | 5 | 130 |
