# Supplementary material for: Indications for bi-cruciate retaining total knee replacement: An international survey of 346 knee surgeons
Source: PLoS One. 2020 Jun 15;15(6):e0234616. doi: 10.1371/journal.pone.0234616 (PMC7295230; doi:10.1371/journal.pone.0234616)
Supplement: S3 File — Supplemental table. (DOCX) [file pone.0234616.s003.docx]

**Digital Supplemental Content 3**

**Experienced ACL intactness and suitability for BCR implantation.**

|  | *Percentage of TKA patients with intact ACL at time of surgery* | | | | | |  |
| --- | --- | --- | --- | --- | --- | --- | --- |
| *Percentage of patients indicated for a knee replacement suitable for BCR* | *None* | *1 - 20%* | *21 - 40%* | *41 - 60%* | *61 - 80%* | *More than 80%* | **Total** |
| None | 3 (0.9%) | 5 (1.4%) | 11 (3.1%) | 10 (2.9%) | 11 (3.1%) | 21 (6.0%) | 61 (17.7%) |
| 1 - 20% | 2 (0.6%) | 18 (5.2%) | 40 (11.6%) | 45 (13.0%) | 46 (13.2%) | 22 (6.3%) | 173 (50.0%) |
| 21 - 40% | 1 (0.3%) | 2 (0.6%) | 8 (2.3%) | 22 (6.3%) | 16 (4.7%) | 9 (2.7%) | 58 (16.8%) |
| 41 - 60% | 0 (0%) | 1 (0.3%) | 2 (0.6%) | 13 (3.8%) | 11 (3.1%) | 10 (2.9%) | 37 (10.7%) |
| 61 - 80% | 0 (0%) | 1 (0.3%) | 1 (0.3%) | 3 (0.9%) | 4 (1.2%) | 8 (2.3%) | 17 (4.9%) |
| More than 80% | 0 (0%) | 0 (0%) | 0 (0%) | 0 (0%) | 0 (0%) | 0 (0%) | 0 (0%) |
| **Total** | 6 (1.8%) | 27 (7.8%) | 62 (17.9%) | 93 (26.9%) | 88 (25.4%) | 70 (20.2%) | 346 (100%) |
