# Supplementary material for: Indications for bi-cruciate retaining total knee replacement: An international survey of 346 knee surgeons
Source: PLoS One. 2020 Jun 15;15(6):e0234616. doi: 10.1371/journal.pone.0234616 (PMC7295230; doi:10.1371/journal.pone.0234616)
Supplement: S4 File — (DOCX) [file pone.0234616.s004.docx]

**S4. ACL assessment and ACL status**

| **Which of the following examinations do you consider reasonable before implanting a BCR TKA?** | *Reasonable* | | *Not reasonable* | | *I do not know* | |
| --- | --- | --- | --- | --- | --- | --- |
|  | *n* | *%* | *n* | *%* | *n* | *%* |
| Magnetic resonance imaging | 232 | 67.1% | 87 | 25.1% | 27 | 7.8% |
| Clinical test (pre-operative) | 311 | 89.9% | 25 | 7.2% | 10 | 2.9% |
| Clinical test (intra-operative) | 299 | 86.4% | 37 | 10.7% | 10 | 2.9% |
| Macroscopic exam (intra-operative) | 293 | 84.7% | 38 | 11.0% | 15 | 4.3% |
| Arthroscopy | 72 | 20.8% | 254 | 73.4% | 20 | 5.8% |

***Note: HCPs can select multiple answers***

| **Which of the following macroscopic appearances of the ACL would you consider eligible for a BCR TKA?** | *N* | *%* |
| --- | --- | --- |
| ACL intact | 320 | 92.5% |
| ACL partially intact | 136 | 39.3% |
| ACL ruptured | 10 | 2.9% |
| ACL missing | 4 | 1.2% |

***Note: HCPs can select multiple answers***
