# Supplementary material for: Indications for bi-cruciate retaining total knee replacement: An international survey of 346 knee surgeons
Source: PLoS One. 2020 Jun 15;15(6):e0234616. doi: 10.1371/journal.pone.0234616 (PMC7295230; doi:10.1371/journal.pone.0234616)
Supplement: Attachment — Submitted filename: 2020-04-24 PONE Point by Point.docx [file pone.0234616.s006.docx]

PONE-D-19-34268
Indications for bi-cruciate retaining total knee replacement: an international survey of 346 knee surgeons

**Point by point rebuttal letter**

Dear Yifei Wang,

Thank you for the review of our manuscript. Please find the following tabular point by point discussion of all comments. A revised manuscript with marked changes and a clean version are attached to the revision per your request.

We thank you and the reviewers for the comprehensive review and believe the revised manuscript has been significantly improved based on your constructive comments.

**Editorial comments:**

| **COMMENT** | **ANSWER** |
| --- | --- |
| We would appreciate receiving your revised manuscript by 6-11-2020. When you are ready to submit your revision, log on to <https://www.editorialmanager.com/pone/> and select the 'Submissions Needing Revision' folder to locate your manuscript file. | n/a |
| If you would like to make changes to your financial disclosure, please include your updated statement in your cover letter. | The financial disclosures have been updated and the cover letter was revised. |
| To enhance the reproducibility of your results, we recommend that if applicable you deposit your laboratory protocols in protocols.io, where a protocol can be assigned its own identifier (DOI) such that it can be cited independently in the future. For instructions see: [http://journals.plos.org/plosone/s/submission-guidelines#loc-laboratory-protocols](https://nam03.safelinks.protection.outlook.com/?url=http%3A%2F%2Fjournals.plos.org%2Fplosone%2Fs%2Fsubmission-guidelines%23loc-laboratory-protocols&data=02%7C01%7Ckolja.boese%40smith-nephew.com%7C04a2bf8a818540cf7ae708d7dfb70177%7C273106dc287842ebb7c8069dcf334687%7C0%7C1%7C637223849412339019&sdata=ggERIaB56XjhS7kkkt10bGdZP0%2BzgjCx31dVKp%2BsMTE%3D&reserved=0) | No laboratory protocol available. The complete questionnaire has been added as supplemental data (**S1**). |
| Please include the following items when submitting your revised manuscript:   - A rebuttal letter that responds to each point raised by the academic editor and reviewer(s). This letter should be uploaded as separate file and labeled 'Response to Reviewers'. - A marked-up copy of your manuscript that highlights changes made to the original version. This file should be uploaded as separate file and labeled 'Revised Manuscript with Track Changes'. - An unmarked version of your revised paper without tracked changes. This file should be uploaded as separate file and labeled 'Manuscript'. | All three documents are attached. |
| 1. Please ensure that your manuscript meets PLOS ONE's style requirements, including those for file naming. The PLOS ONE style templates can be found at  <http://www.journals.plos.org/plosone/s/file?id=wjVg/PLOSOne_formatting_sample_main_body.pdf> and <http://www.journals.plos.org/plosone/s/file?id=ba62/PLOSOne_formatting_sample_title_authors_affiliations.pdf> | The templates are now being used. The manuscript has been changed accordingly. |
| 2. Please include additional information regarding the survey or questionnaire used in the study and ensure that you have provided sufficient details that others could replicate the analyses. For instance, if you developed a questionnaire as part of this study and it is not under a copyright more restrictive than CC-BY, please include a copy, in both the original language and English, as Supporting Information." | The complete questionnaire (S1; English and German version) has been added as supplemental data.  Please note, that a supplemental Excel file (S5) is attached. It includes all individual answers by participants for complete transparency. |
| 3. Please provide additional details regarding participant consent. In the ethics statement in the Methods and online submission information, please ensure that you have specified (1) whether consent was informed and (2) what type you obtained (for instance, written or verbal, and if verbal, how it was documented and witnessed). If the need for consent was waived by the ethics committee, please include this information. | All participants gave written (online web-form) consent. No personal information was collected. The survey was anonymous.  The Methods Section has been updated.  The online submission information has been updated. |
| Thank you for stating the following in the Competing Interests section: "I have read the journal's policy and the authors of this manuscript have the following competing interests: CKB, DDF and MS are employed by Smith & Nephew."  We note that one or more of the authors have an affiliation to the commercial funders of this research study : "Smith + Nephew"  a) Please provide an amended Funding Statement declaring this commercial affiliation, as well as a statement regarding the Role of Funders in your study. If the funding organization did not play a role in the study design, data collection and analysis, decision to publish, or preparation of the manuscript and only provided financial support in the form of authors' salaries and/or research materials, please review your statements relating to the author contributions, and ensure you have specifically and accurately indicated the role(s) that these authors had in your study. You can update author roles in the Author Contributions section of the online submission form.  Please also include the following statement within your amended Funding Statement.  “The funder provided support in the form of salaries for authors [insert relevant initials], but did not have any additional role in the study design, data collection and analysis, decision to publish, or preparation of the manuscript. The specific roles of these authors are articulated in the ‘author contributions’ section.”  If your commercial affiliation did play a role in your study, please state and explain this role within your updated Funding Statement.  b). Please also provide an updated Competing Interests Statement declaring this commercial affiliation along with any other relevant declarations relating to employment, consultancy, patents, products in development, or marketed products, etc.  Within your Competing Interests Statement, please confirm that this commercial affiliation does not alter your adherence to all PLOS ONE policies on sharing data and materials by including the following statement: "This does not alter our adherence to  PLOS ONE policies on sharing data and materials.” (as detailed online in our guide for authors [http://journals.plos.org/plosone/s/competing-interests](https://nam03.safelinks.protection.outlook.com/?url=http%3A%2F%2Fjournals.plos.org%2Fplosone%2Fs%2Fcompeting-interests&data=02%7C01%7Ckolja.boese%40smith-nephew.com%7C04a2bf8a818540cf7ae708d7dfb70177%7C273106dc287842ebb7c8069dcf334687%7C0%7C1%7C637223849412344013&sdata=6dhofMPUOjN3Lm8JtAZuTHWl5VcvPpn6koahyq%2FSIbI%3D&reserved=0)). If this adherence statement is not accurate and  there are restrictions on sharing of data and/or materials, please state these. Please note that we cannot proceed with consideration of your article until this information has been declared.  Please include both an updated Funding Statement and Competing Interests Statement in your cover letter. We will change the online submission form on your behalf.  Please know it is PLOS ONE policy for corresponding authors to declare, on behalf of all authors, all potential competing interests for the purposes of transparency. PLOS defines a competing interest as anything that interferes with, or could reasonably be perceived as interfering with, the full and objective presentation, peer review, editorial decision-making, or publication of research or non-research articles submitted to one of the journals. Competing interests can be financial or non-financial, professional, or personal. Competing interests can arise in relationship to an organization or another person. Please follow this link to our website for more details on competing interests: [http://journals.plos.org/plosone/s/competing-interests](https://nam03.safelinks.protection.outlook.com/?url=http%3A%2F%2Fjournals.plos.org%2Fplosone%2Fs%2Fcompeting-interests&data=02%7C01%7Ckolja.boese%40smith-nephew.com%7C04a2bf8a818540cf7ae708d7dfb70177%7C273106dc287842ebb7c8069dcf334687%7C0%7C1%7C637223849412349007&sdata=F0iw3LWy5X4JzSLIUcO700%2Bf%2FTONnSX1hVpZ2U87SVc%3D&reserved=0) | The statement was added to the competing interests section.   a) An amended Funding Statement has been added.   The following statement has been added:  “The study was funded by Smith + Nephew as an investigator initiated study (IIS 585). The funder provided support in the form of coverage of costs associated with the survey (fee for online services by SurveyMonkey), coverage of publication fees (if applicable) and salaries for authors CKB, DDF and MS, but did not have any additional role in the study design, data collection and analysis, decision to publish, or preparation of the manuscript. The specific roles of these authors are articulated in the ‘author contributions’ section.”   b) Not required   Statement has been added.  "This does not alter our adherence to PLOS ONE policies on sharing data and materials.”  The cover letter has been updated accordingly. |
| 5. Please include captions for your Supporting Information files at the end of your manuscript, and update any in-text citations to match accordingly. Please see our Supporting Information guidelines for more information: [http://journals.plos.org/plosone/s/supporting-information](https://nam03.safelinks.protection.outlook.com/?url=http%3A%2F%2Fjournals.plos.org%2Fplosone%2Fs%2Fsupporting-information&data=02%7C01%7Ckolja.boese%40smith-nephew.com%7C04a2bf8a818540cf7ae708d7dfb70177%7C273106dc287842ebb7c8069dcf334687%7C0%7C1%7C637223849412353996&sdata=6fphK0gcDtVB1EB1kPifyZ9sZ5Sx8qLNuOFNUyxNPnI%3D&reserved=0). | The manuscript has been updated to cover this requirement. |

**Reviewers' comments:

Reviewer's Responses to Questions**

| **COMMENT** | **ANSWER** |
| --- | --- |
| 1. Is the manuscript technically sound, and do the data support the conclusions?  The manuscript must describe a technically sound piece of scientific research with data that supports the conclusions. Experiments must have been conducted rigorously, with appropriate controls, replication, and sample sizes. The conclusions must be drawn appropriately based on the data presented.   Reviewer #1: Yes  Reviewer #2: Yes | Nothing to add. |
| 2. Has the statistical analysis been performed appropriately and rigorously?   Reviewer #1: Yes  Reviewer #2: Yes | Nothing to add. |
| 3. Have the authors made all data underlying the findings in their manuscript fully available?  The [PLOS Data policy](https://nam03.safelinks.protection.outlook.com/?url=http%3A%2F%2Fwww.plosone.org%2Fstatic%2Fpolicies.action%23sharing&data=02%7C01%7Ckolja.boese%40smith-nephew.com%7C04a2bf8a818540cf7ae708d7dfb70177%7C273106dc287842ebb7c8069dcf334687%7C0%7C1%7C637223849412358987&sdata=llPxwj4kCMSmvHh1%2FpBYnv09p9MUpQ0gZkFuKS69fss%3D&reserved=0) requires authors to make all data underlying the findings described in their manuscript fully available without restriction, with rare exception (please refer to the Data Availability Statement in the manuscript PDF file). The data should be provided as part of the manuscript or its supporting information, or deposited to a public repository. For example, in addition to summary statistics, the data points behind means, medians and variance measures should be available. If there are restrictions on publicly sharing data—e.g. participant privacy or use of data from a third party—those must be specified.  Reviewer #1: Yes  Reviewer #2: Yes | Nothing to add. |
| 4. Is the manuscript presented in an intelligible fashion and written in standard English?  PLOS ONE does not copyedit accepted manuscripts, so the language in submitted articles must be clear, correct, and unambiguous. Any typographical or grammatical errors should be corrected at revision, so please note any specific errors here.  Reviewer #1: Yes  Reviewer #2: Yes | Nothing to add. |

**Comments to the Author**

| Reviewer #1: This is a very useful survey based report of surgeons' opinions toward BCR TKA. The survey went through a formation process and the statistical analysis is well done and analyzed in a way that helps understand not only the surgeons' responses but how their practices correlate with their opinions on BCR TKA. Cleary the authors are suggesting in the paper that surgeons may be willing to embrace BCR TKA with more information and practice. To that end, I have the following suggestions for improvement: | We thank the reviewer for the positive comments and overall appraisal of our study. |
| --- | --- |
| 1) The introduction and discussion present citations for the concepts of knee kinematics and difficulty of procedure as they relate to the opinions presented in the study. However, many surgeons may not embrace BCR TKA because of the lack of difference in objective functional outcomes or implant survival making it worth embracing a new more difficult technique. In addition to the study cited in lines 286-288, the discussion should have a citation regarding the survival rates of BCR TKA, and in addition to the citation in 284-85 regarding the forgotten knee sensation, an additional note should be made about the objective functional outcomes of UKA/BCR TKA compared to traditional TKA | The reviewer highlights the relevance of clinical outcome data on BCR TKA. In particular, objective as well as patient reported outcomes and survival data.  The discussion section was expanded and relevant information from a non-published systematic literature review on clinical outcomes of BCR TKA was included.  Of note, there is only limited clinical evidence on BCR TKA. In particular, for novel BCR TKA designs, long-term data is not yet available.  “Survival analysis of old BCR TKA designs with follow-up of up to 24 years reached 89% (82-93%) in a study by Pritchett. For modern designs, the follow-up times were shorter and results were heterogeneous. While Alnachoukati et al. reported 1.4% at 12 months, Baumann et al. found revision rates of up to 5.9% at 18 months and, Pelt et al. 17% revisions for any reason at 36 months for the same implant type.”  “They found BCR TKA and UKA to have comparable joint awareness in terms of the forgotten joint score (BCR TKA 53.4 vs. UKA 53.6 points). Both were superior to the standard TKA group (38.9 points) yet no objective knee scores were reported.(2, 9)” |
| 2) The surgeons' experience in number of cases is presented, but the training may be an important component of surgeons' opinions as well. Perhaps sports fellowship trained physicians would be more likely to embrace this technique than joints fellowship trained physicians. | The reviewer indicates the training background of participants. While this potential bias has been discussed during the survey development, more extensive questions on training and background were removed during early stages of the questionnaire. Multiple factors of training and education of the participating surgeons could and will have influences the answers. However, a more detailed description was deemed too complex. To address your comment, we have added a paragraph in the limitations of the discussion section. |
| 3) In the sections regarding an intact ACL (290-296), how was this determined? MRI, clinical exam, evaluation during surgery, or combination of the 3? Clearly the decision should be made prior to surgery if one is choosing to perform BCR TKA so it would be useful to have an objective measure of how this was determined | The comment addresses a most important aspect of planning BCR TKA. Generally, a functionally intact ACL is a prerequisite for BCR TKA.  Participants were asked three specific questions regarding ACL in BCR TKA.  Q30: What percentage of your TKA patients do you feel have an intact ACL at the time of surgery?  Q31: Which of the following examinations do you consider reasonable before implanting a BCR TKA?   - Magnetic resonance imaging (MRI) - Clinical test (Pre-operative) - Clinical test (Intra-operative) - Macroscopic exam (intra-operative) - Arthroscopy - Other   Q32: Which of the following macroscopic appearances of the ACL would you consider eligible for a BCR TKA?   - ACL intact - ACL partially intact (e.g. fraying, degenerative changes, etc.) - ACL ruptured - ACL missing   Additionally, Q33 and Q34 asked about single- and two-stage ACL reconstruction.  Q30 results reported on experience of intraoperative findings. Therefore, it is something of a baseline for expectations in future patients. Supplemental material S2 and S3 provide further insight into this question.  Q31 refers to the reviewer’s question. Here, participants were asked to specify what pre-operative test may be required to identify functional intact ACL. The results of this question were added to the results section and a paragraph was added to the discussion section.  “Participants would apply MRI (67%, n=232) or a pre-operative clinical function test (90%, n=311) of the ACL to determine the ACL status (S4). 136 participants (39%) would indicate BCR TKA in ACL with signs of degeneration (S4).”  “In this survey, the majority of surgeons (90%) considered pre-interventional clinical function tests and 67% MRI to be to be reasonable.”  Two tables with detailed results were added as Supplemental Information S4 covering Q31 and Q32. |
| 4) Given that the findings of this study are based on a theoretical adaptation of a surgical technique by a group of surgeons, was there any follow up to see if some actually embraced the concept and performed BCR TKA after the survey? Particular attention, or possible future study should look at surgeons who were not performing BCR TKA who then began performing the procedure, and evaluating why they switched, as well as comparing matched results from their patients undergoing the different procedures. If this can't be performed and included here, a comment on plans to perform that study may strengthen the impact of the current study. | The reviewer raises an interesting question. Per protocol, the survey was collected anonymously and therefore no follow-up with the participants was possible. Therefore, no matching of results can be performed.  Given the idea, we have start internal conversations to consider developing a study to address this interesting research project.  Thank you for bringing this to our attention. |
| 5) In the section discussing contracture/deformity, reasons suggesting why one would use a border for too much deformity are presented. An additional possibility is that deformity beyond 10 degrees in either direction likely requires soft tissue release(s) for knee balancing that will require additional options for constraint not presented when performing BCR TKA. Perhaps a line commenting on the lack of constraint options is required here. | The reviewer’s comment on contractures and deformities is important. We have changed the manuscript accordingly.  “These limitations may reflect the lack of constraint in BCR TKA which reduce the options of soft tissue balancing.” |
| 6) A section is dedicated to discussing ACL competence. Is there a reported ACL rupture rate requiring re-operation following BCR TKA that could be included here as surgeons weigh the possibility of switching to this procedure? | A review of the literature on BCR did not show ACL rupture following BCR TKA leading to revision surgery. |
| Overall this is a useful snapshot assessing how surgeons may approach whether to adapt a new procedure, with appropriate references for the reasoning behind adapting the new procedure presented in a coherent manner, strengthened by the acknowledged limitation that this is a survey based study of surgeons' opinions rather than a presentation of a fact-based argument. | Thank you again for the comprehensive review. |
| Reviewer #2: General comments:  The authors have surveyed TKA surgeons including those who currently perform bicruicate raining TKA and those who do not, regarding their indications for TKA which retains both cruciate ligaments. Surgeons who perform UKA or CR TKA were found to more likely indicate BCR than surgeons who routinely sacrifice both cruciate ligaments during TKA. The study also found some consensus among surgeons regarding appropriate indications for the procedure. Bicruciate retaining TKA has been done in the past and was abandoned largely resulting from mechanical failure of the tibial construct. | We thank the reviewer for the constructive comments. |
| It would be helpful to compare the indications for modern bicruciate TKA’s in the current study to those used in the past since more narrow indications may affect the durability of the procedure. | The reviewer comments on the indications for BCR TKA. Of note, a recent non-published systematic literature analysis on BCR TKA by our workgroup was not able to identify clear indications for BCR TKA. The lack of reliable information on indications was one of the major reasons for the present survey.  The information gained from the systematic literature review informed the development of the survey and fed into the scope of questions.  Please find the **attached table** at the end of this document showing the limited information extracted from clinical studies. |
| Introduction, last paragraph. What was the study hypothesis? | This survey aimed to (1) identify interest in and acceptance of BCR surgery and (2) to capture the range of indications for BCR in the opinion of practicing knee surgeons. Given the descriptive nature of the study, no specific hypothesis was formulated.  It may be said that the H_0_ is “There is no interest in BCR TKA by surgeons experienced in total knee arthroplasty”. However, due to the descriptive design, we refrained from putting a hypothesis in the manuscript. |
| Materials and Methods, first paragraph. What were the fundamental areas of questioning included in the survey such as age, BMI, deformity, gender, and activity level, and why were they considered important? Why were two languages (English and German used for the survey)? | The survey aimed to define the perceived indications and indication limitations of BCR TKA from the perspective of experience knee joint replacement surgeons.  The fundamental areas are (separated by categories):  Baseline information on participants and experience (gender, job position, experience (TKA, partial knees), PCL sacrificing)  General on BCR TKA (interest in, experience with, suitable patients, expected advantages)  Specific indication of BCR TKA (etiology/extent of OA, age, BMI, contracture, deformity)  For details please refer to the complete questionnaire added as **supplemental information S1**  The selection of fundamental areas was based on:   - a systematic review on BCR TKA - internal review process with orthopaedic surgeons - pilot survey with experienced external TKA surgeons including expert panels   A bi-lingual survey was developed to include the German speaking countries (Germany, Austria, Switzerland) without language restrictions. |
| Materials and Methods, Survey design. The specific survey questions or most relevant ones should be included in a table or appendix. | The complete survey (German and English) has been added as **supplemental material S1** |
| Results. Were there any significant differences in reposes between the German and English speaking surgeons? | While – per protocol - no statistical analysis on this was performed, we did not notice any relevant differences between German and English survey answers. Additionally, both language options were available for all participants and therefore it is possible that answers of both survey languages include participants from other regions. |
| Results. Were there any significant differences in indications for surgeons who performed bicruciate retaining TKA and those without bicruciate retaining TKA experience? | The reviewer raises an important comment. In the analysis, we compared indications between BCR TKA experienced and unexperienced surgeons. While a detailed presentation was deemed to be too extensive as part of this manuscript, we have highlighted the information in the discussion section:  “However, previous experience with BCR TKA influenced the limitations regarding upper BMI, upper age, contractures and varus deformity. In all cases, experienced surgeons were less restrictive.”  However, due to the large number of variables and the associated high risk of creating alpha errors, we refrained from performing statistical tests and reporting p-values. We still feel the provided information in combination with supplemental raw data of all answers suffices for adequate interpretation of the data. |
| Discussion, page 16. Are there any differences in surgeons’ indications for currently available modern bicruciate retaining TKA’s compared with those that had been used in the past and were abandoned? | The questionnaire did not specifically asked about specific implants but rather the general willingness and indication spectrum of BCR TKA. Therefore, no differences between generations of BCR TKA can be analysed. Only few surgeons were experienced with old generations of BCR TKA (i.e. LCS; n=7). However, due to the small number, no subgroup analysis was performed. |
| Discussion, line 313. Some prior studies of earlier bicruciate retaining TKA’s (Pritchett, Cloutier) showered good survivorship. Were the indications used in prior studies of successful BCR similar or different than the authors current survey findings? | The literature on indications for BCR TKA is very limited as has been found in a non-published systematic literature review of BCR TKA performed before development of the survey. Pritchett as well as Cloutier did only report very limited information on indications. Thus, a detailed comparison of indications cannot be performed.  Additional, please refer to the table below for reference of available information on indications in previous clinical studies on BCR TKA. |
| Tables. Tables 7 and 8 appear to contain information which is summarized adequately in the text and should be removed. | We thank the reviewer for the thorough analysis of our manuscript.  Please note that tables 7 and 8 do show additional information on indications with specific separation of data by two questions:   1) surgeons preference regarding PCL sacrifice 2) general willingness to perform BCR TKA   We feel both tables add to a better understanding of the ranges of indications and do not show redundant data. |

**Supportive Table from systematic literature review showing reported indications for BCR TKA.**

| Author* | Year | Indications | | | |
| --- | --- | --- | --- | --- | --- |
|  |  | Etiology | Deformity | Contracture | Soft tissue |
| Alnachoukati | 2018 | + |  |  | + |
| Baumann | 2017 | + | Valgus <10°  Varus <10° |  |  |
| Buechel | 1990 |  | + |  |  |
| Christensen | 2017 | + | + (minimal deformity) | + |  |
| Cloutier | 1991 | OA; RA; ON | Valgus <30°  Varus <30° |  |  |
| Cloutier | 2001 | OA; RA | Valgus 11-20°  Varus 1-30° | 25-65° |  |
| Lavoie | 2018 | + (no limitations) | + (indirect) | + |  |
| Pelt | 2019 | OA; RA; revision | Valgus <15°  Varus <15° | < 15° |  |
| Pritchett | 1996 |  | + (no severe deformity) |  |  |
| Pritchett | 2004 |  | Valgus>15°  Varus>20° | > 20° |  |
| Pritchet | 2013 |  |  |  |  |
| Pritchett | 2015 | OA; RA; ON | Valgus <15°  Varus <15° |  |  |
| Sabouret | 2013 | OA; RA |  |  |  |
| Stiehl | 2006 | OA; RA; PT; OT |  |  |  |
| Tsai | 2019 | Varus OA |  |  |  |

Abbreviations: OA – Osteoarthritis; RA – rheumatoid (inflammatory) arthritis; ON – osteonecrosis; PT – post-traumatic; OT – other.

+ indicates mentioning of indications without specification.
